# An Electronic Patient-Reported Outcome Tool for the FACT-B (Functional Assessment of Cancer Therapy-Breast) Questionnaire for Measuring the Health-Related Quality of Life in Patients With Breast Cancer: Reliability Study

**DOI:** 10.2196/10004

**Published:** 2019-01-22

**Authors:** Lina Maria Matthies, Florin-Andrei Taran, Lucia Keilmann, Andreas Schneeweiss, Elisabeth Simoes, Andreas D Hartkopf, Alexander N Sokolov, Christina B Walter, Nina Sickenberger, Stephanie Wallwiener, Manuel Feisst, Paul Gass, Michael P Lux, Florian Schuetz, Peter A Fasching, Christof Sohn, Sara Y Brucker, Joachim Graf, Markus Wallwiener

**Affiliations:** 1 Hospital for General Obstetrics and Gynecology Gynecologic Oncology, National Center for Tumor Diseases University Hospital Heidelberg Heidelberg Germany; 2 Department of Women’s Health University Hospital Tübingen Tübingen Germany; 3 Research Institute for Women’s Health Department of Women’s Health University Hospital Tübingen Tübingen Germany; 4 Section of Midwifery Science Institute for Health Sciences University Hospital Tübingen Tübingen Germany; 5 Institute for Medical Biometry and Informatics University Hospital Heidelberg Heidelberg Germany; 6 Department of Gynecology and Obstetrics University Breast Center Franconia, Comprehensive Cancer Center Erlangen-EMN University Hospital Erlangen Erlangen Germany

**Keywords:** breast cancer, ePRO measurement, FACT-B, HRQoL, patient-reported outcomes, reliability of ePRO

## Abstract

**Background:**

The most frequent malignant disease in women is breast cancer. In the metastatic setting, quality of life is the primary therapeutic goal, and systematic treatment has only a limited effect on survival rates; therefore, the concept of the health-related quality of life (HRQoL) and measurement of patient-reported outcomes (PROs) are gaining more and more importance in the therapy setting of diseases such as breast cancer. One of the frequently used questionnaires for measuring the HRQoL in patients with breast cancer is the Functional Assessment of Cancer Therapy-Breast (FACT-B). Currently, paper-based surveys still predominate, as only a few reliable and validated electronic-based questionnaires are available. ePRO tools for the FACT-B questionnaire with proven reliability are missing so far.

**Objective:**

The aim of this study was to analyze the reliability of tablet-based measurement of FACT-B in the German language in adjuvant (curative) and metastatic breast cancer patients.

**Methods:**

Paper- and tablet-based questionnaires were completed by a total of 106 female adjuvant and metastatic breast cancer patients. All patients were required to complete the electronically based (ePRO) and paper-based version of the FACT-B. A frequency analysis was performed to determine descriptive sociodemographic characteristics. Both dimensions of reliability (parallel forms reliability using Wilcoxon test and test of internal consistency using Spearman ρ) and agreement rates for single items, Kendall tau for each subscale, and total score were analyzed.

**Results:**

High correlations were shown for both dimensions of reliability (parallel forms reliability and internal consistency) in the patients’ response behavior between paper-based and electronically based questionnaires. Regarding the reliability test of parallel forms, no significant differences were found in 35 of 37 single items, while significant correlations in the test for consistency were found in all 37 single items, in all 5 sum individual item subscale scores, as well as in total FACT-B score.

**Conclusions:**

The ePRO version of the FACT-B questionnaire is reliable for patients with breast cancer in both adjuvant and metastatic settings, showing highly significant correlations with the paper-based version in almost all questions all subscales and the total score.

## Introduction

### Breast Cancer: Epidemiological Relevance

The most frequent malignant disease in women is breast cancer; indeed, about 70,000 new cases of breast cancer are diagnosed in Germany every year. Therapeutic options have been improved, resulting in an overall 5-year survival rate of patients with early-stage disease of >90% [1-3]. In contrast, the prognosis of metastatic breast cancer is significantly poorer, since palliative treatment often remains the only option due to the low probability of cure in patients with metastatic disease [4,5].

### Patient-Reported Outcomes and Health-Related Quality of Life in Patients With Breast Cancer

Since systematic palliative treatment has only a limited effect on survival rates, the concept of the health-related quality of life (HRQoL) and measurement of patient-reported outcomes (PROs) are gaining more and more importance in the therapy setting of progressive diseases, such as breast cancer, especially in the adjuvant or metastatic setting [6-10]. Patients with cancer often suffer from symptoms and adverse events during their treatment, which are sometimes underestimated in clinical routine [11-13]. Although clinicians’ perception of symptoms can predict unfavorable clinical events more precisely, patients´ reports can reflect the daily health status more adequately [14]. PROs comprise various aspects of the subjectively perceived state of health from the patients’ point of view such as HRQoL, satisfaction with care, and drug adherence [9,15,16]. With regard to the therapy setting, monitoring HRQoL and the occurrence of symptoms appears to be of particular relevance, primarily during therapy, but also as a long-term follow-up for improving and supporting patients´ well-being [17-20]. The importance of measuring PRO in patients with breast cancer is also stated in the German S3-guideline [21]. As (metastatic) breast cancer often remains an incurable disease with only palliative treatment options, monitoring the HRQoL is highly relevant in these patients [4,22,23]. Different questionnaires highlight different aspects of symptoms and HRQoL [24]. One of the frequently used questionnaires for measuring the HRQoL in patients with breast cancer is the Functional Assessment of Cancer Therapy-Breast (FACT-B), a validated, multidimensional questionnaire with 37 items that build 5 dimensions (subscales) when using a 5-point Likert scale [25-27].

### Electronic Measurement of Patient-Reported Outcomes

Collecting and analyzing pencil and paper-based data are difficult tasks without possibilities for direct response or interaction [15]; therefore, the electronic-based measurement of PRO (ePRO) is gaining more and more importance. There are several advantages of ePRO such as rapid access to data, a probable avoidance of errors during data entry, fewer missing data in comparison with paper-based surveys, the capacity to trigger alerts or notifications for answers to special circumstances, and an improvement in patients’ willingness to report sensitive information [23,28]. Although paper-based surveys of PRO still predominate because there are only a few reliable and validated ePRO questionnaires, numerous projects have evaluated the feasibility and acceptance of HRQoL in the ePRO measurement in the last few years [29-34]. Nevertheless, knowledge regarding patients’ acceptance, feasibility, and barriers remains limited [35], especially because hurdles might exist in relation to health status, technical skills, and socioeconomic aspects, which could influence both patients´ willingness to use ePRO and their response behavior [10,36,37]. Although studies have already demonstrated a potential equivalence between some paper-based PRO and ePRO, the reliability of ePRO questionnaires should be verified so as not to endanger the validity of ePRO surveys [10,36-40]. Indeed, ePRO tools for the FACT-B questionnaire with proven reliability are missing so far.

### Aims and Objectives

The aim of the study was to analyze the reliability of a tablet-based ePRO app for FACT-B in German for measuring the HRQoL in adjuvant and metastatic breast cancer patients in comparison with the validated paper-based version of FACT-B. It was planned to determine whether differences exist in response behavior between the validated paper-based PRO version of FACT-B and a new ePRO version, whether the answers between paper-based and ePRO questionnaire differ in a relevant way, and whether the patients’ response behavior is influenced by the mode of answering (paper- or tablet-based). In order to achieve these aims, patients were asked to complete both the paper- and tablet-based version of the FACT-B questionnaire.

## Methods

### Sample and Study Design

From July 2015 to May 2016, paper-based and tablet-based PRO questionnaires were completed by a total of 106 female adjuvant and metastatic breast cancer patients treated consecutively at the Department of Women’s Health (Tübingen, Germany) and the National Cancer Center (Heidelberg, Germany). Patients were recruited as a part of the electronic Patient-Reported Outcomes and Compliance Analysis (ePROCOM) and Patient Engagement Pilotstudie Mammakarzinom-individualisierte und Ressourcen-effiziente Patient Reported Outcomes Erfassung durch Digitale Therapieunterstützungssysteme (PEPPER) studies. The aims of ePROCOM were to evaluate the general patient acceptance and practicability of a Web-based app for a PRO questionnaire for patients with adjuvant or metastatic breast cancer. Patients were asked to participate to compare their response behavior in paper-based and Web-based questionnaires and analyze the reliability of the ePRO versions of the questionnaires European Organisation for Research and Treatment of Cancer (EORTC) quality of life questionnaire (QLQ)-C30, as published previously [40], and FACT-B (reported in this paper). The PEPPER study intends to evaluate the impact of Web-based and paper-based PRO for health care services by patients from the Prospective Academic Translational Research Network for the Optimization of the Oncological Health Care Quality in the Adjuvant and Advanced/Metastatic Setting: Health Care Research, Pharmacogenomics, Biomarkers, Health Economics study. The methods are described in detail in the EORTC paper [40]. The inclusion criteria of the ePROCOM and PEPPER studies were female gender, full legal age, the proven diagnosis of breast cancer in adjuvant or metastatic setting, sufficient language skills in German, and signed declaration of consent. The exclusion criterion was participation in other studies to minimize the burden of questionnaires. Patients were asked to complete the questionnaire during an outpatient visit at the hospital under the supervision of an attending physician. The study was designed as a 2-center (Tübingen and Heidelberg), 2-armed, prospective randomized trial. All patients were required to complete both the electronically based (ePRO) and paper-based version of the FACT-B HRQoL questionnaire. Patients in arm A were assigned to start with the tablet version followed by the paper version in the same session. Patients in arm B filled out the paper-based version followed by the tablet-based questionnaire. The randomization procedure was based on permuted-block randomization, which strives to generate equally large treatment groups [41,42]. The postexposure acceptance for using the ePRO tool was high (92%) as patients were asked whether they could potentially imagine using tablet-based tools before using ePRO [37]. Patients were informed about the aims of this study and were asked for their consent *ex ante*. The study was approved by the Ethics Committee at the University of Tübingen (project number 089/2015B02) [40].

### Procedure

**Figure 1:**
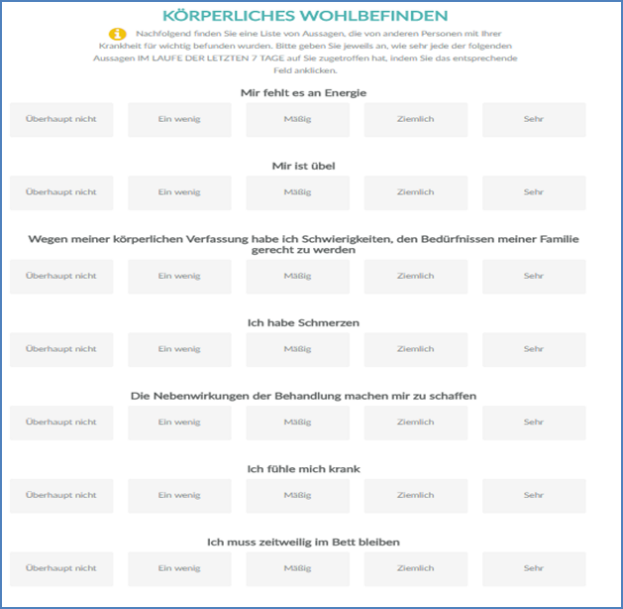
Screenshot of the Patient-informiert-interaktiv-Arzt app’s FACT-B (Functional Assessment of Cancer Therapy-Breast) questionnaire for the dimension “physical well-being” (German). Source: Authors work, licensed under fair use.

The data collection was performed in 4 parts. The first part focused on patientsʼ socioeconomic variables. The second part comprised the FACT-B questionnaire, consisting of 37 questions with responses required on a 5-point Likert scale (from 0, *not at all* through 4, *very much*) that constitute 5 dimensions [25-27,43]. The response options labels (*not at all* through *very much*; Figure 1) were the same in both the standard German paper-based and the tablet-based versions. While in the third part of the assessment, patients were asked about preexisting technical skills, their willingness to use ePRO, and potential barriers in relation to their health status [37], the fourth part concerned with the patients’ evaluation of the ePRO tool (manuscript in preparation). All patients completed the second part of the assessment both on paper and using a tablet, while they answered the questions in the other parts only in paper-based form. In this study, we report the results of the second part of the assessment (reliability analysis of the ePRO tool of FACT-B) [40].

### Specifics of the Electronic Patient-Reported Outcomes Tool

For measuring ePRO, we used the “Patient-informiert- interaktiv-Arzt (PiiA),” that is “patient interactively informs doctor” Web-based app, which allows patients to answer the relevant questions on a tablet. The PiiA portal is a Web-based solution for capturing PROs, which our working group has developed. Patients receive anonymized user credentials and are asked to complete the FACT-B questionnaire. Figure 1 shows the user interface of the first set of questions of the German FACT-B. After completing the questionnaires, patients log out and their pseudoanonymized data are backed up on a local storage device and securely locked [40].

### Statistical Analyses

All statistical analyses were conducted using SPSS Statistics (IBM, version 24). First, a frequency analysis was performed to determine the descriptive sociodemographic characteristics of patients. After that, we analyzed both dimensions of reliability (parallel forms reliability and test of internal consistency) and examined the disparity of responses and the rate of consistency between paper- and tablet-based responses. Both types of reliability were calculated for the 37 single items as well as for scores of the 5 dimensions, including the subscales for Physical Well-Being (PWB), Social/Family Well-Being (SWB), Emotional Well-Being (EWB), Functional Well-Being (FWB), and Breast Cancer Subscale (BCS), and the FACT-B total score in accordance with the FACT-B guidelines [25-27]. According to the Shapiro-Wilks test, the paired samples were not normally distributed; therefore, we used the Wilcoxon test to identify possible statistically significant differences in the *test of parallel forms reliability* both between the single items and the scores. Initially, the mean values of the paper- and tablet-based measures were calculated according to the official FACT-B guidelines [25-27]. Second, the consistency analyses were performed by calculation of Spearman rank correlation coefficient (Spearman ρ) and agreement rates for every FACT-B item along with rank correlation (Kendall tau) for each scale. Prior to that, we performed chi-square tests and Shapiro-Wilks test comparing metastatic and adjuvant breast cancer patients to identify possible statistically significant differences in relation to HRQoL. In all analyses, *P*<.05 (2-tailed) was considered indicative of statistically significant differences (alpha=.05).

As such an analysis is considered an explorative study, all reported *P* values can be taken as purely descriptive. Missing values (which arose when patients did not answer individual questions) were appropriately taken into account in the calculation of the scores that were ignored in the statistical calculation [40]. Both figures (boxplot and correlation diagram) were generated in SPSS 24.

## Results

### Patient Enrollment

**Figure 2:**
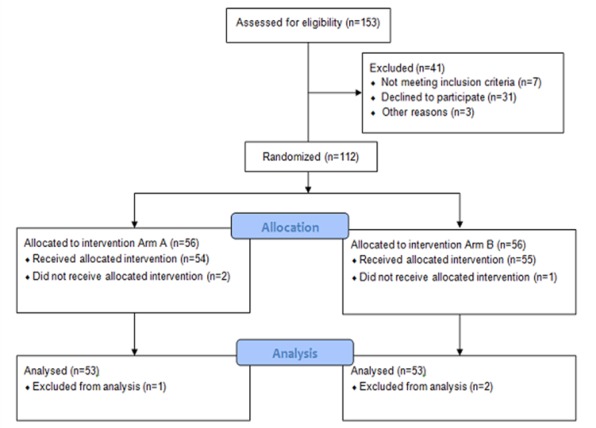
Consolidated Standards of Reporting Trials flow diagram.

Overall, 106 eligible female patients with breast cancer were recruited who completed questions from FACT-B both in a paper-based format and electronically using a tablet. Originally, 153 patients were assessed for eligibility, of which 47 were excluded during recruiting, allocation, and data analysis, as shown in the Consolidated Standards of Reporting Trials flow diagram (Figure 2).

In all, 53 patients were assigned to tablet-based filling followed by paper-based filling in the same session (arm A), while the same number of patients completed the paper-based version followed by the tablet-based questionnaire (arm B). Both the paper- and tablet-based questionnaires were completed by patients consecutively during the same ambulance visit. Patients who had not completed more than half of the FACT-B questions in either paper- or tablet-based format were excluded (arm A, 1 patient; arm B, 2 patients). We did not find any significant differences between the 2 arms in the response behavior, sociodemographic status, or therapy setting; therefore, the 2 arms were considered together. Beforehand, all single items of the 2 arms were compared. Ten patients (arm A) and 16 patients (arm B), respectively, produced missing data in some questions (more often in the tablet-based questionnaire) [40].

### Sociodemographic Variables

Tables 1 and 2 shows the sociodemographic characteristics of the study group, with 71.6 (76/106) of patients in adjuvant therapy and 28.3% (30/106) in a metastatic situation. We did not find any marked intergroup differences between metastatic and adjuvant breast cancer patients in either ePRO or paper-based PRO. Although adjuvant and metastatic patients differed in age and HRQoL, as metastatic patients were older and reported a poorer HRQoL, we found no differences in the ePRO response behavior between the 2 groups. In addition, there were no differences in the reliability in any of the single items and scales between metastatic and adjuvant patients; hence, the complete patient collective was considered together. The mean age of the whole collective was 51.0 years; nearly one-third of the patients had a higher level of education (high school diploma) [40].

**Table 1:** Sociodemographic characteristics of the patients (n=106).

Sociodemographic variables		Descriptive analyses
**Age**		
	Mean (SD)	51 (11.31)
	Median (range, minimum-maximum)	52 (54, 30-84)
**Level of education^a^**		
	Median	3
	Interquartile range (25%-75% quantiles)	2 (3-5)

^a^Level of education: 1=lowest; 5=highest.

**Table 2:** Education level and therapy setting of the patients (n=106).

Variable		Frequency, n (%)	95% CI
**Level of education**			
	No qualification	1 (0.9)	0-6
	Main or secondary school graduation	43 (40.5)	32-50
	Advanced technical graduation	19 (17.9)	10-26
	High school diploma^a^	33 (31.1)	22-40
	Not specified	10 (9.4)	2-15
**Therapy setting**			
	Metastatic	30 (28.3)	19-35
	Adjuvant setting	76 (71.6)	61-83

^a^High school diploma indicates “Abitur.”

### Parallel Forms Reliability

Table 3 presents the results of the Wilcoxon test for analyzing parallel forms reliability in the single items of FACT-B. The ePRO tool seems to demonstrate acceptable parallel forms reliability as only 2 significant differences (out of 37 in total) could be found in the single-item comparison. A weak statistically significant difference could only be identified in questions GP 4 (*I have pain*) and GS 2 (*I get emotional support from my family*). The pain reports were slightly higher in the ePRO questionnaire (*P*=.012), while emotional support was evaluated somewhat higher in paper-based PRO (*P*=.036). The differences were only slight, though, as the medians of the 2 items could not be distinguished from each other. In 35 of 37 questions in the FACT-B, no statistically significant differences were observed between patients’ answers in the paper-based questionnaire and ePRO.

In addition, slight differences were noted between paper-based PRO and ePRO in the individual item scores of the 5 dimensions and the total FACT-B score (Table 4). The differences were significant in 2 dimensions (EWB and BCS) but not in the total scores because the scores included several questions (PWB, SWB, and FWB: 7 questions; EWB: 6 questions; BCS: 10 questions), whereby differences in patients’ responses to single questions could be multiplied in the scores and missing values caused fluctuations between the paper- and tablet-based PRO. The statistical differences in 2 scores, therefore, appear to be of methodological origin rather than attributable to differences in the response behavior. As only 5 response options (0-4) are available for each question in the FACT-B questionnaire, the ranking procedure often results in a large number of ties in the Wilcoxon test, which produces larger *P* values.

**Figure 3:**
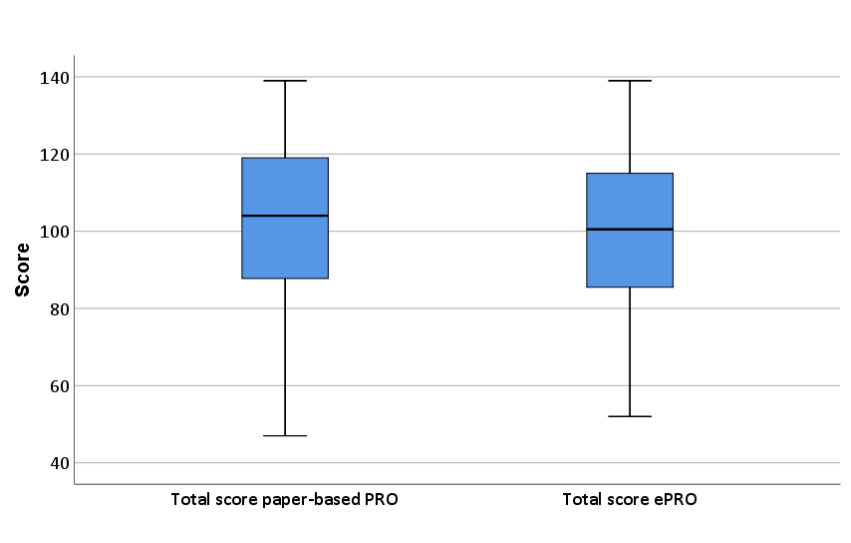
Distribution of total scores (Boxplot). PRO: patient-reported outcome.

The total score is slightly higher in ePRO (mean difference: 1.73; median difference: 0.63), but without statistically significant differences. Figure 3 shows the distribution of the paper-based and ePRO total score for FACT-B in a boxplot. It is obvious that the whisker but not the interquartile range is broader in the paper-based version.

**Table 3:** Parallel forms reliability (Wilcoxon test) in single items.

Single items		Paper-based patient-reported outcomes		Electronic patient-reported outcomes		*P* value	
		Mean (SD)	Median (Interquartile range)	Mean (SD)	Median (Interquartile range)		
**Physical Well-Being**							
	GP1	1.68 (1.22)	2 (2)	1.59 (1.21)	1 (1)		.22
	GP2	0.54 (0.86)	0 (1)	0.60 (0.88)	0 (1)		.26
	GP3	1.34 (1.21)	1 (2)	1.29 (1.18)	1 (2)		.72
	GP4	0.98 (1.01)	1 (1.25)	1.03 (0.99)	1 (2)		*.01* ^a^
	GP5	1.50 (1.17)	1 (1)	1.58 (1.09)	1 (1)		.08
	GP6	1.22 (1.12)	1 (2)	1.23 (1.05)	1 (2)		.24
	GP7	0.77 (1.09)	0 (1)	0.73 (1.03)	0 (1)		>.99
**Social/Family Well-Being**							
	GS1	3.20 (0.94)	3 (1)	3.16 (1.04)	3 (1)		.82
	GS2	3.61 (0.73)	4 (1)	3.5 (0.86)	4 (1)		*.04* ^a^
	GS3	3.18 (1.09)	4 (1)	3.14 (1.1)	3 (1)		>.99
	GS4	3.33 (0.75)	3 (1)	3.28 (0.81)	3 (1)		.39
	GS5	3.37 (0.82)	4 (1)	3.33 (0.77)	3 (1)		.11
	GS6	3.65 (0.77)	4 (.0)	3.63 (0.82)	4 (1)		.83
	GS7	1.98 (1.66)	2 (2.5)	2.07 (1.11)	2 (2)		.72
**Emotional Well-Being**							
	GE1	1.32 (1.09)	1 (1)	1.22 (1.01)	1 (1)		.40
	GE2	2.68 (1.16)	3 (2)	2.91 (0.98)	3 (2)		.05
	GE3	0.60 (1.17)	0 (1)	0.44 (0.77)	0 (1)		.11
	GE4	1.19 (1.03)	1 (2)	1.12 (0.99)	1 (2)		.83
	GE5	1.22 (1.14)	1 (2)	1.18 (1.05)	1 (1.5)		.70
	GE6	1.42 (1.26)	1 (1)	1.32 (1.06)	1 (1)		.59
**Functional Well-Being**							
	GF1	2.12 (1.22)	1 (1)	2.21 (1.20)	1 (1)		.23
	GF2	2.30 (1.20)	2 (2)	2.32 (1.12)	2 (2)		.34
	GF3	2.50 (1.10)	3 (1)	2.51 (1.11)	3 (1)		.81
	GF4	2.58 (1.04)	3 (1)	2.55 (1.01)	3 (1)		.39
	GF5	2.40 (1.18)	3 (1)	2.41 (1.15)	3 (1)		.25
	GF6	2.53 (1.15)	3 (1)	2.65 (1.05)	3 (1)		.20
	GF7	2.19 (1.15)	2 (1)	2.17 (1.07)	2 (1.5)		.81
**Breast Cancer Subscale**							
	B1	0.75 (0.93)	0.25 (1)	0.72 (0.90)	0.5 (1)		.49
	B2	0.58 (1.10)	0 (1)	0.52 (0.99)	0 (1)		.79
	B3	0.68 (1.03)	0 (1)	0.63 (0.91)	0 (1)		>.99
	B4	1.73 (1.04)	2 (1)	1.69 (1.01)	2 (1)		.81
	B5	1.47 (1.41)	1 (2)	1.47 (1.38)	1 (2)		.49
	B6	1.88 (1.41)	2 (2)	1.82 (1.45)	1.5 (2)		.06
	B7	2.02 (1.38)	2 (2)	2.14 (1.33)	2 (2)		.33
	B8	1.23 (1.34)	1 (2)	1.13 (1.37)	1 (2)		.11
	B9	2.55 (1.18)	3 (1)	2.62 (1.17)	3 (2)		.58
	P2	1.28 (1.11)	1 (2)	1.13 (1.05)	1 (1.75)		.18

^a^Statistically significant difference.

**Table 4:** Parallel forms reliability (Wilcoxon test) for scoring values of 5 Functional Assessment of Cancer Therapy-Breast (FACT-B) dimensions (subscales).

FACT-B^a^ dimensions	FACT-B questions	Paper-based patient-reported outcomes		Electronic patient-reported outcomes		*P* value
		Mean (SD)	Median (Interquartile range)	Mean (SD)	Median (Interquartile range)	

Physical Well-Being Sum individual item scores	GP1-GP7	19.97 (6.11)	21.0 (9.0)	19.89 (5.88)	20.0 (8.25)	0.05
Social/Family Well-Being Sum individual item scores	GS1-GS7	22.88 (3.93)	24.0 (5.0)	22.34 (4.60)	23.33 (5.0)	0.25
Emotinal Well-Being Sum individual item scores	GE1-GE6	16.89 (4.84)	18.0 (6.0)	17.73 (4.68)	18.0 (6.0)	.*01*^*b*^
Functional Well-Being Sum individual item scores	GF1-GF7	16.73 (6.29)	18.0 (9.0)	16.75 (6.10)	18.0 (9.45)	0.69
Breast Cancer Subscale Sum individual item scores	B1-B9; P2	26.35 (6.28)	28.0 (9.0)	28.56 (7.11)	30.0 (11.33)	<.*001*^*b*^
FACT-B total score		102.66 (22.0)	106.33 (28.81)	104.39 (22.47)	107 (30.31)	0.05

^a^FACT-B^:^ Functional Assessment of Cancer Therapy-Breast

^a^Statistically significant difference.

### Test of Internal Consistency

Table 5 shows the Spearman ρ correlation values and agreement rates, which were obtained for every question of the FACT-B questionnaire. In 27 questions, a high correlation (>.8) was found between paper-based PRO and ePRO, while the correlation was moderate (>.5) in the other 10 questions. In 20 questions, the correlation levels amounted to >.85. In all 37 correlated questions, agreement rates fluctuated between 65.7% and 91.8%.

**Figure 4:**
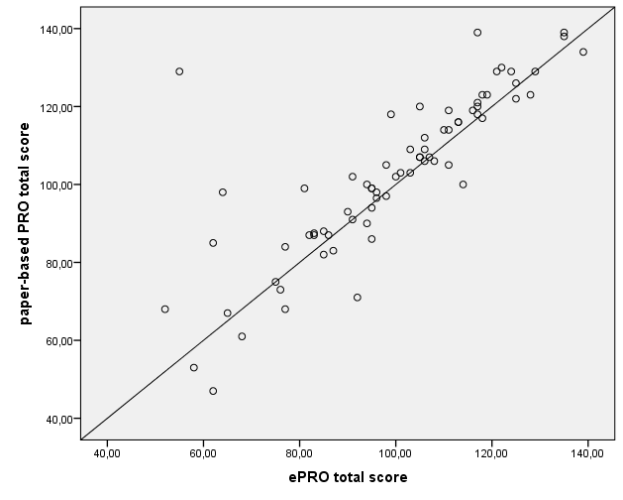
Correlation between electronic patient-reported outcome (ePRO) and paper-based total Functional Assessment of Cancer Therapy-Breast scores.

Table 6 shows the results of internal consistency testing for the sum individual item subscale scores and the total FACT-B score between paper-based PRO and ePRO. In all functional (sub)scales, the rank correlation was moderate to high as Kendall tau coefficient ranged between.64 and.80 in the sum individual item subscale scores and amounted to.80 in the total FACT-B score. The analysis of internal consistency tests showed the highest correlation in the sum individual item subscale scores EWB and FWB. All results of internal consistency tests were statistically highly significant. For illustrative purposes, Figure 4 represents a strong positive correlation between the ePRO and paper-based total FACT-B scores. Each data point reflects individual scores of patients.

**Table 5:** Test of internal consistency in single items: results of correlation (Spearman *ρ*) and agreement analyses.

Dimensions		Spearman ρ	*P* value of Spearman ρ^a^	Agreement (%)
**Physical Well-Being**				
	GP1	0.869	<.001	72.0
	GP2	0.836	<.001	86.9
	GP3	0.866	<.001	70.1
	GP4	0.836	<.001	75.4
	GP5	0.837	<.001	68.3
	GP6	0.842	<.001	71.5
	GP7	0.889	<.001	86.4
**Social/Family Well-Being**				
	GS1	0.782	<.001	77.8
	GS2	0.880	<.001	85.4
	GS3	0.908	<.001	87.8
	GS4	0.782	<.001	76.3
	GS5	0.829	<.001	81.3
	GS6	0.931	<.001	91.8
	GS7	0.747	<.001	77.0
**Emotional Well-Being**				
	GE1	0.753	<.001	71.0
	GE2	0.525	<.001	66.6
	GE3	0.796	<.001	82.3
	GE4	0.868	<.001	78.0
	GE5	0.931	<.001	85.8
	GE6	0.733	<.001	65.7
**Functional Well-Being**				
	GF1	0.881	<.001	74.0
	GF2	0.770	<.001	69.7
	GF3	0.889	<.001	81.0
	GF4	0.821	<.001	71.5
	GF5	0.934	<.001	87.2
	GF6	0.910	<.001	82.4
	GF7	0.897	<.001	75.2
**Breast Cancer Subscale**				
	B1	0.858	<.001	86.4
	B2	0.925	<.001	87.0
	B3	0.904	<.001	87.9
	B4	0.777	<.001	80.7
	B5	0.900	<.001	80.6
	B6	0.949	<.001	83.6
	B7	0.843	<.001	69.5
	B8	0.945	<.001	86.0
	B9	0.684	<.001	70.1
	P2	0.850	<.001	73.8

^a^Statistically highly significant correlations.

**Table 6:** Test of internal consistency in the individual subscale item scores and the total score: Kendall tau analysis.

Dimensions	Kendall tau (95% CI)	*P* value of Kendall tau^a^
Physical Well-Being Sum individual item scores	0.784 (0.723-0.835)	<.*001*
Social/Family Well-Being Sum individual item scores	0.648 (0.545-0.749)	<.*001*
Emotional Well-Being Sum individual item scores	0.737 (0.638-0.820)	<.*001*
Functional Well-Being Sum individual item scores	0.797 (0.731-0.858)	<.*001*
Breast Cancer Subscale Sum individual item scores	0.638 (0.536-0.724)	<.*001*
Total score	0.801 (0.741-0.852)	<.*001*

^a^Statistically significant correlations.

## Discussion

### Principal Results

In both dimensions of reliability (parallel forms reliability and internal consistency), we found high correlations when comparing single items in the patients’ response behavior between paper-based PRO and ePRO in the FACT-B questionnaire. In the test of parallel forms reliability, we only found statistically significant differences in all but 2 questions. In the test of consistency, moderate to high correlations were found in all 37 single items and all sum individual item subscale scores. Based on the empirical results, the PiiA tool’s ePRO version of FACT-B seems to be reliable for measuring the HRQoL in patients with metastatic and adjuvant breast cancer. According to these results, we would not expect that future use of the PiiA tool in the *same* patient group will show marked differences between the paper-based PRO and ePRO version of FACT-B or that patients’ response behavior is significantly influenced by the survey tool after transfer to electronically based patient surveys. Therefore, the tool is suitable for determining the HRQoL in patients with metastatic or adjuvant breast cancer.

### Comparison With Prior Work

Although ePRO apps are being used increasingly frequently, paper-based surveys of PRO still predominate in clinical research because reliable, electronically validated questionnaires are lacking. One of the most commonly used questionnaires for measuring the HRQoL, especially in patients with breast cancer, is the FACT-B, for which there is a reliable, paper-based format in many languages but no reliable, electronically based version exists in German. Using the electronically based version of FACT-B and other PRO without verifying the reliability could endanger the significance of ePRO surveys. Thus, a corresponding analysis in relation to differences and correlations between the paper-based version of FACT-B and newly developed Web-based tools are of great importance. It can be assumed that many aspects (ie, sociodemographic aspects, technical skills, health condition, and, perhaps, design specifics of the ePRO tool) may influence both patients’ willingness to use the tool and their response behavior, which underlines the need for reliability analyses [10,24,35-37]. However, almost no scientific studies have dealt with the reliability of ePRO questionnaires. Now, our working group has verified the reliability of an ePRO version of the FACT-B questionnaire in this paper, as well as the reliability of a tool for the ePRO measurement of the HRQoL in the EORTC questionnaire [40].

### Limitations

Despite positive results, some limitations of the study design and methodological implementation should be mentioned, which could reduce the representativeness of the data. In 2 of 37 questions, we found statistically significant differences by the Wilcoxon test (parallel forms reliability). This can be a random observation based on numerous tests performed. A further explanation could be that patients were less concentrated owing to time pressure. Patients (both in arms A and B) were required to complete both paper-based and ePRO during an outpatient hospital visit. Patients were surveyed while they were receiving chemotherapy and were not permitted to take the questionnaire home to complete it; this also explains increasing missing values, especially in the last quarter of both questionnaire versions; possibly the length of the survey (paper-based and ePRO FACT-B and EORTC QLQ C-30, socioeconomic data, and evaluation of the tool) was too extensive for some patients. Possibly, the burden of disease and the therapy were potentially affecting the ability of some patients to complete both the paper- and tablet-based version of the questionnaire during an outpatient visit. Hence, a possible limiting factor was an inadequate screening as to whether all patients were able to cope with the psychological burden of participating in the study, as it is known that psycho-oncological distress is a commonly associated burden that could potentially influence the willingness to use ePRO and as a result ePRO’s reliability [37,44]. Nonetheless, the influence of psycho-oncological stress was likely low in this study, as the test of internal consistency showed no abnormalities. Both the individual questions and the sum individual item subscale scores showed consistently statistically significant correlations. It also needs to be noted that there was possibly a selection bias, as we did not examine whether the HRQoL was lower and the psychological distress was higher in those patients who could not be motivated to participate in the study. Therefore, it cannot be claimed with certainty that the tool *per se* is reliable for all patients with metastatic and adjuvant breast cancer; hence, further studies are needed that focus on the willingness to use ePRO depending on the state of health [10].

### Strengths of the Study

Although ePRO is being used more and more often, questionnaires with proven reliability and validity are lacking. FACT-B is one of the most commonly used questionnaires worldwide for measuring the HRQoL in patients with breast cancer, but a reliable ePRO version is also missing here. One of the strengths of this study is that the reliability of a new tool for the ePRO measurement in patients with breast cancer was analyzed, while other studies often assign paper-based versions to a tablet-based format without verifying the reliability. The reliability of the PiiA tool could be ascertained for the questionnaires FACT-B (this paper) and EORTC QLQ C-30 [40] for measuring the HRQoL in metastatic and adjuvant breast cancer patients. The second strength is the methodological approach of the study ePROCOM and the statistical evaluation as all patients completed both the paper-based and ePRO questionnaire, and the reliability was ascertained in a multidimensional fashion (parallel forms reliability as well as internal consistency). Finally, the third strength points to the fact that ePRO tools are reliable and suitable in both the adjuvant and metastatic settings, although hurdles can be expected in these patient groups, in particular, depending on their health status, HRQoL, sociodemographic specifics, and technical ability and experience [10,37]. The results of this study can improve the quality of ePRO measurements as they seem to be generalizable, and the PiiA app of FACT-B can be used for reliable e-based measurement of the HRQoL in other studies and clinical routine.

### Conclusions

Electronically based PRO is constantly being adopted in clinical research and clinical routine, which underlines the need for reliable questionnaires. The evaluated electronic version of the FACT-B is reliable for patients with breast cancer in an adjuvant or metastatic setting because high correlations were found in almost all questions, all subscales, and the total score. Thus, this study concludes that the validated paper-based questionnaire of FACT-B and the ePRO tool are equal.

## Appendix

Multimedia Appendix 1 CONSORT-EHEALTH checklist (V 1.6.1).

## Abbreviations

BCS: Breast Cancer Subscale
ePROCOM: electronic Patient-Reported Outcomes and Compliance Analysis
EORTC: European Organisation for Research and Treatment of Cancer
EWB: Emotional Well-Being
FACT-B: Functional Assessment of Cancer Therapy-Breast
FWB: Functional Well-Being
HRQoL: health-related quality of life
PEPPER: Patient Engagement Pilotstudie Mammakarzinom - individualisierte und Ressourcen-effiziente Patient Reported Outcomes Erfassung durch Digitale Therapieunterstützungssysteme
PiiA: Patient-informiert-interaktiv-Arzt
PRO: patient-reported outcomes
PWB: Physical Well-Being
QLQ: quality of life questionnaire
SWB: Social/Family Well-Being
